# Influence of Wine pH and Ethanol Content on the Fining Efficacy of Proteins from Winemaking By-Products

**DOI:** 10.3390/foods11121688

**Published:** 2022-06-08

**Authors:** Berta Baca-Bocanegra, Sandra Gonçalves, Julio Nogales-Bueno, Inês Mansinhos, Francisco José Heredia, José Miguel Hernández-Hierro, Anabela Romano

**Affiliations:** 1 Department of Analytical Chemistry, Facultad de Farmacia, Universidad de Sevilla, 41012 Sevilla, Spain; bbaca1@us.es (B.B.-B.); julionogales@us.es (J.N.-B.); 2 MED—Mediterranean Institute for Agriculture, Environment and Development and CHANGE–Global Change and Sustainability Institute, Faculdade de Ciências e Tecnologia, Universidade do Algarve, Campus de Gambelas, Ed. 8, 8005-139 Faro, Portugal; smgoncalves@ualg.pt (S.G.); ifmansinhos@ualg.pt (I.M.); aromano@ualg.pt (A.R.); 3 Food Colour and Quality Laboratory, Department of Nutrition and Food Science, Facultad de Farmacia, Universidad de Sevilla, 41012 Sevilla, Spain; heredia@us.es

**Keywords:** red wine, grape seed protein, winemaking by-product, fining, phenolic compounds, antioxidant activity, color

## Abstract

Wine color and limpidity are important aspects of consumer preferences. The alteration of these parameters can damage wine’s appearance but also its mouthfeel characteristics due to its relationship with attributes such as bitterness and astringency. Fining is a practice usually used in enology to modulate undesirable wine organoleptic attributes. However, there are several factors that influence this technique. In this study, the influence of wine pH and ethanol content on grape seed protein fining efficacy has been assessed. Wine clarification, total phenolic and flavanol contents, antioxidant activity, and chromatic parameters have been investigated before and after fining process. The most noticeable clarifying effects were observed for the experimental wines with a lower pH and ethanol content. Control of these factors will make it possible to modulate the main organoleptic properties of the wine, also avoiding the addition of large amounts of fining agents and thus providing greater versatility to wineries during winemaking. Furthermore, our findings indicated that grape seed protein is a potential alternative to other plant-based fining proteins commonly used in winemaking. Its effects on clarification and color quality have been found to be comparable to those of potato protein and significantly better than those of pea protein.

## 1. Introduction

Sensory attributes such as color, limpidity, bitterness, and astringency are commonly used by consumers to evaluate the quality of wines, especially red wines. Color and limpidity are important aspects of visual satisfaction since it is the first attribute perceived by the consumers. Pale colors, the presence of unexpected tones according to the age of the wine, or the presence of particles in suspension lead to the rejection of the product by the consumer. The alteration of these parameters will damage not only the appearance of the wine but also its mouthfeel characteristics due to its relationship with other attributes such as bitterness and astringency [1,2,3]. Phenolic compounds are important for high-quality red wine and play a key role in its organoleptic properties [4]. Hence, changes in the sensory attributes of the wines are connected to qualitative or quantitative variations of the wine phenolic compounds. Further related to their phenolic composition, red wines have been shown to have important biological effects such as antioxidant activity [5,6].

Natural clarification occurs during the winemaking process, leading to the precipitation of particles in suspension. However, this is often not enough, and the remaining unstable compounds can interact with the coloring matter and with the colorless phenolic compounds, leading to the formation of aggregates that modify the wine’s sensory properties and, consequently, their quality [7]. Thus, the control of particles in suspension is a major concern in wineries and an important research line in enology aiming the prevention of visual, flavor, or color alterations, without leading to the loss of beneficial properties of wines, such as their antioxidant capacity, before wine bottling and consumption.

Fining is a practice commonly used in enology to improve the stability of wine by removing or reducing the presence of components responsible for undesirable properties by adding a reactive or adsorptive material [7,8,9]. The interaction between fining agent and phenolic compounds modulates the color of the wines due to pigments precipitation [10], while the interaction with tannins leads to modifications in astringency and bitterness [11,12]. A high number of fining agents have been used during the winemaking procedure, especially those of proteinaceous origin, due to the high capacity of proteins to interact with phenolic compounds [13,14]. Animal proteins such as bovine and porcine gelatin, ovalbumin, or casein revealed good clarity effectiveness. However, the health hazard potential of these proteins, the increase in legal limitations, and the high demand for vegan/vegetarian and natural wines has boosted the evaluation of the fining efficacy of vegetal proteins and non-proteinaceous materials, with promising results [15,16,17,18,19,20]. In recent years, research lines have been directed toward the use of fining agents obtained from materials that are naturally present in grapes, wine, or yeast, thus avoiding the use of exogenous substances and the application of specific legislation for their use [21,22,23]. In this sense, the fining efficacy of grape seed protein extracts obtained from winemaking by-products has been evaluated [24,25,26].

Different fining agents can have a positive or negative effect on the sensory attributes, stability, and composition of the wine. As a result, smooth and elegant wines or, conversely, rougher and less attractive wines can be obtained after the fining stage. Characteristics of the fining agent such as type, hydrolysis degree, doses, and contact time play a fundamental role in the final attributes of the wine [8,11,12,22,27]. Nevertheless, factors directly related to the wine, such as the type, age and composition, and chemical characteristics of the matrix, also influence the final product [9,27,28,29]. The wine characteristics are highly variable, especially in terms of phenolic composition, pH, and concentration of ethanol and sugars. Though until now, studies that evaluate the efficacy of grape seed proteins have focused only on the impact of fining doses and grape variety, as well as their comparison with other proteins of animal and vegetable origin commonly used in winemaking [24,25,26]. In the case of red warm climate wines, which usually have insufficient levels of phenolics, fining process and all the factors that condition it represents a special challenge since an excess of clarification could have a negative impact on the stabilization processes of those compounds directly related to color.

The aim of this research was to assess the influence of wine pH and ethanol content on the fining efficacy of grape seed proteins and their impact on the quality of warm climate red wines in advanced stages of vinification. Wine clarification, total phenolic and flavanol contents, antioxidant activity, and chromatic parameters have been investigated before and after fining process. Moreover, grape seed proteins obtained as a winery by-product were evaluated as a potential alternative to other vegetal proteins (protein isolates from potato and pea) commonly used as fining agents in winemaking.

## 2. Materials and Methods

### 2.1. Fining Agents

Grape seed protein (GSP) was isolated according to the optimized procedure described in Baca-Bocanegra et al. [30]. Briefly, defatted grape seed meal (industrial wine by-product, ALVINESA Natural Ingredients, Ciudad Real, Spain) was subjected to alkaline extraction followed by isoelectric precipitation. For that, pH = 10, T^a^ = 36 °C, 1:9 meal/solvent ratio and 2 h extraction time were used. Precipitated proteins were freeze dried and stored in airtight containers until use. Commercial protein isolates of potato (PROVEGET FINE, *Solanum tuberosum* L.) and pea (PROVEGET 100, *Pisum sativum* L.) provided from Agrovin S.A. (Ciudad Real, Spain) were used in this study for comparison. The Kjeldahl method was applied to determine the protein content of the fining agents, being 79.92 ± 0.21, 72.54 ± 0.39, and 55.35 ± 0.10% for the potato, pea, and grape seed isolate, respectively.

### 2.2. Wine Elaboration and Fining with Grape Seed Protein

With the aim of evaluating the effect of the wine characteristics on the fining efficacy of grape seed protein, a full factorial design with two independent variables at three levels, commonly found in wines, was performed. Wine pH (3.3, 3.6 and 3.9) and ethanol content (11, 13 and 15% *v*/*v*) were evaluated as independent variables. A total of 9 wines with different pH values and ethanol content were elaborated in duplicate using a Syrah red wine 10 months after fermentation as base wine (Table 1).

The base red wine was made from grapes *Vitis vinifera* L. var. Syrah grown in ‘‘Condado de Huelva” Designation of Origin (DO), in the southwest of Spain (warm climate). The characteristics of the base Syrah wine determined according to the official methods described by the European Union are summarized in Table 2.

The pH modification of the experimental wines was carried out using NaOH or HCl solutions, while the alcohol content of the wine was altered using deionized water or 96% ethanol. The fining agent (potato, pea, or grape seed isolate) was added in a proportion of 10 g/hL, using 200 mL glass bottles as containers, and kept in the dark for 6 days at room temperature. An untreated sample of each wine was used as a control. After 6 days of clarification, wine samples were subjected to different analytical determinations. Clarification, total phenolic content, total flavanolic contents, antioxidant activity, and color were measured as a response variable in the factorial design.

### 2.3. Clarification

The absorption spectra of wine samples comprised between 350 and 850 nm were recorded in triplicate at constant intervals (Δλ = 2 nm) with an Agilent 8453 UV–Vis spectrophotometer (Palo Alto, CA, USA), using glass cells of 10 mm path length. Taking into account the spectral characteristics of red wines in the measured region, for comparative purposes, it can be considered that from 750 nm, the absorption changes vs. a reference without the fining agent could be mainly related to the reduction of the particles and compounds in suspension and also the dispersion phenomenon. In view of these circumstances, the spectral region between 750 and 850 nm, specifically its area under the curve, was used to evaluate the changes in turbidity of the wines due to the addition of the fining agents. For each experimental red wine, the clarification has been evaluated as the percentage of absorbance reduction with respect to that of its corresponding control.

### 2.4. Total Phenolic Contents

A modification of the Folin–Ciocalteu method described by Ainsworth and Gillespie [31] was used for the determination of total phenolic content (TPC). Briefly, 100 µL of each wine sample were mixed in triplicate with 200 µL of 10% (*v*/*v*) Folin–Ciocalteu reagent and 800 µL of 700 mM Na_2_CO_3_. After 2 h at room temperature, the absorbance of the mixture was measured at 765 nm in a Tecan-Infinite M200 microplate reader. A calibration curve of gallic acid was used for quantification, and the results were expressed as gallic acid equivalents in mg/L of wine.

### 2.5. Total Flavanol Content

A slight modification of the protocol reported by Vivas et al. [32] was used for measuring the total flavanol contents (TFC) in the wine samples. For that, 20 µL of wine sample, 180 μL of methanol, and 1 mL of DMACA (4-(Dimethylamino)cinnamaldehyde) reagent (0.1% *w*/*v* in a mixture of HCl: methanol 1:10, *v*/*v*) were mixed and incubated for 10 min. After the incubation period, the mixture absorbance was measured at 640 nm on a UV–visible spectrophotometer. The total flavanol content was calculated as (+)–catechin equivalents in mg/L of wine using the regression equation between (+)–catechin standards and the measured absorbance.

### 2.6. Antioxidant Activity

The antioxidant activity of all clarified wines and their respective controls was evaluated using 2,2-diphenyl-1-picrylhydrazyl (DPPH free radical scavenging), 2,2′-azino-bis (3-ethylbenzothiazoline-6-sulfonic acid) diammonium salt (ABTS•+ radical cation decoloration), Ferric reducing antioxidant power (FRAP), and Oxygen Radical Absorbance Capacity (ORAC) methods. In all assays, three independent replicates were performed for each sample.

#### 2.6.1. DPPH Free Radical Scavenging Assay

The ability of the wine samples to scavenge DPPH radicals was evaluated following the method previously reported by Soler-Rivas et al. [33]. For that, 30 μL of wine sample, 300 μL of 90 μM DPPH methanolic solution, and 570 μL of methanol were mixed. Using a spectrophotometer, the absorbance of the mixture was measured at 515 nm after an incubation period of 30 min in the dark and at room temperature. The radical scavenger activity was expressed as milligrams of (±)-6-hydroxy-2,5,7,8-tetramethylchromane-2-carboxylic acid (Trolox) equivalents per liter of wine (mgTE/L wine).

#### 2.6.2. ABTS•+ Radical Cation Decoloration Assay

The antioxidant activity was also assessed based on the generation of the ABTS radical cation (ABTS•+) and the measurement of its decay as a consequence of the addition of an antioxidant-containing wine. Following the procedure described by Re et al. [34] potassium persulfate was used as an oxidizing agent to prepare the stock solution of ABTS•+, which was then diluted with phosphate-buffered saline to provide an absorbance of 0.70 ± 0.02 at 734 nm. Then, 190 μL of this reagent was mixed with 10 μL of each wine sample, and the absorbance was measured at 734 nm. The results were expressed as mg of Trolox equivalents per liter of wine (mgTE/L wine).

#### 2.6.3. Ferric Reducing Antioxidant Power (FRAP)

The method reported by Pulido et al. [35] was applied to determine the antioxidant reducing ability of the wine samples. Briefly, a mixture comprising 100 μL of properly diluted wine sample, 250 µL sodium phosphate buffer (200 mM, pH 6.6), and 250 µL 1% K_3_[Fe(CN)_6_] was incubated for 20 min at 50 °C. At the end of the incubation, 250 µL of 10% trichloroacetic acid was added, followed by centrifugation. Then, 400 μL of the obtained supernatant was diluted with the same amount of water and mixed with 80 μL 0.1% FeCl_3_. The reduction activity was determined by reading the absorbance of the mixture at 700 nm. The antioxidant capacity was expressed as mg of ascorbic acid equivalents per liter of wine (mgAAE/L wine).

#### 2.6.4. Oxygen Radical Absorbance Capacity (ORAC) Assay

The oxygen radical absorbance ability of the samples was assessed following the protocol described by Gillespie et al. [36], using fluorescein as the fluorescent probe and 2,2′-azobis(2-methylpropionamidine) dihydrochloride (AAPH) as the peroxyl radical generator. In a black microplate, 150 µL of 0.2 µM fluorescein solution were mixed with 25 µL of sample, and incubated for 10 min at 37 °C. After that, 25 µL of AAPH (150 mM) were added to each well, and fluorescence was measured in a microplate reader every 5 min for 90 min using 485 nm and 530 nm as excitation and emission wavelength, respectively. A quadratic regression equation, obtained from the concentrations of Trolox stock solutions (6.25–50 µM) and the area under the curve (AUC), was applied to calculate the ORAC values. Results were expressed as mg of Trolox equivalents per liter of wine (mgTE/L wine).

### 2.7. Colorimetric Measurements

Using the original software CromaLab^®^, the CIELAB parameters (L*, a*, b*, C*_ab_, and h_ab_) of each wine sample were obtained based on their visible spectra (380–770 nm) and following the recommendations of the Commission Internationale de l‘Éclairage: CIE 1964, 10° standard observer, and the CIE D65 illuminant [37]. Wine color differences (ΔE*_ab_) were determined as the euclidean distance between two points in the three-dimensional space defined by L*, a*, and b*: ΔE*_ab_ = [(ΔL*)^2^ + (Δa*)^2^ + (Δb*)^2^]^1/2^.

### 2.8. Statistical Analysis

Univariate analyses of variance (ANOVA) and Tukey’s post hoc test were applied to establish statistical differences in the mean values obtained for the different parameters evaluated according to the wine pH and ethanol content variables. Following this same procedure, the fining potential of grape seed protein was evaluated against potato and pea protein. The correlation between total phenolic content and antioxidant activity was studied using univariate linear regression. The data were presented as the mean of three replicates of each experiment. All statistical analyzes, including experimental design, were performed using Statistica v.8.0 software (StatSoft Inc., Tulsa, OK, USA, 2007).

## 3. Results and Discussion

### 3.1. Experimental Design for Grape Seed Protein Fining

In this study, the relevance of wine pH and ethanol content during the fining procedure with grape seed protein (GSP) was evaluated owing to the increasing variability found in wines that concern these parameters. According to the full factorial design, GSP was added to nine experimental red wines with different pH values and ethanol contents, and the influence on wine clarification, total phenolic content, total flavanol content, antioxidant activity, and chromatic parameters have been investigated. The effect of each factor and their interaction on the above-mentioned responses are compiled in Table 3. According to the ANOVA of the 3^2^ full factorial design, ethanol content is the most relevant factor for the variables analyzed in this study. This factor, in its linear form, has turned out to be significant for clarification, total phenolics, total flavanols, and the antioxidant activity determined by the ABTS, FRAP, and ORAC methods. A similar effect was observed when studying the factor of relevance pH, except for the flavanol content variable for which this factor did not show a significant contribution. On the other hand, no significant impact of either of the two factors on DPPH scavenging activity and colorimetric parameters has been observed. The quadratic term of the independent variables showed to have a significant influence only for total phenolics. The ANOVA also showed that the interaction between pH and ethanol content has a significant impact only for clarification and antioxidant activity measured with ABTS and FRAP assays.

**Figure 1:**
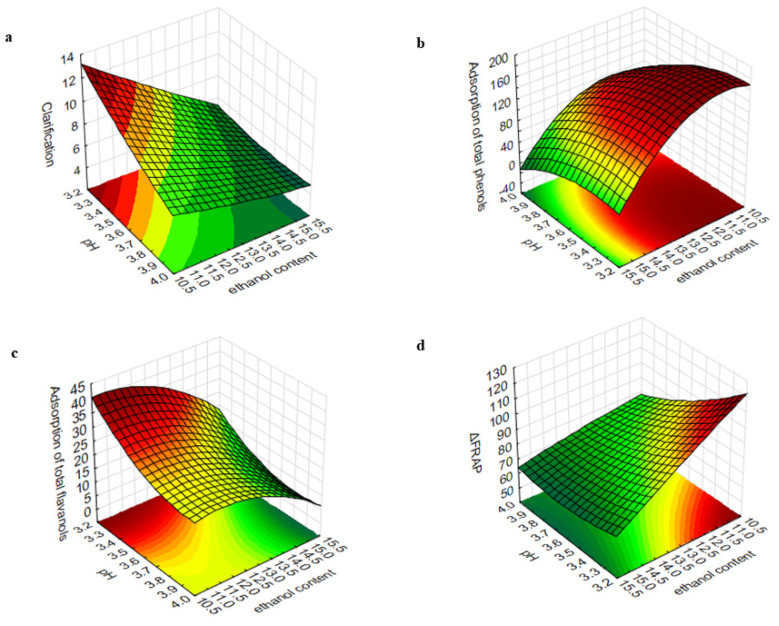
Influence of wine pH and ethanol content on (**a**) clarification (%), (**b**) adsorption of total phenols (mg/L), (**c**) adsorption of total flavanols (mg/L) and (**d**) Δ antioxidant activity determined by FRAP method (mg AAE/L).

The results were also graphically represented as three-dimensional response surfaces to visualize the relative effects of the two independent factors (pH and ethanol content) on dependent variables (Figure 1). In order to visualize the effect on antioxidant activity, the data obtained by the FRAP method have been chosen as an example.

#### 3.1.1. Impact on Clarification

In all samples, fining treatment with grape seed proteins increased the limpidity of the wines (Table 1). The degree of clarification after 6 days of fining treatment ranged from 5.04 to 11.47% under the tested conditions. The sedimentation or precipitation processes that occur naturally in wines during the stabilization period led to a decrease in the high turbidity typical of young wines. Considering that a 10-month aged wine has been used in this study, the generally low impact on the clarification observed in all analyzed wines was expected, in agreement with the results previously reported [9,25].

The clarification model of the 3^2^ full factorial designs was analyzed. The F value in this model for the linear form of pH and ethanol content and their interaction was 247.96 (*p* < 0.001), 715.81 (*p* < 0.001) and 79.89 (*p* < 0.01), respectively. As can be observed in Figure 1a, pH and ethanol content modulate the response, in the same way, exerting negative effects on the clarification, so higher pH, and ethanol content decreased the efficacy of GSP clarification. Hence, the most noticeable clarifying effects were obtained for the experimental wines with a lower pH and ethanol content (Table 1). However, these differences are reduced as the value of the studied factors increases, even ceasing to be significant in some cases. These results show a positive influence of the pH x ethanol interaction on the efficiency of the clarification.

#### 3.1.2. Impact on Total Phenolic and Flavanol Contents

Changes in total phenolic (TPC) and flavanol contents (TFC) were found between fined and unfined red wines. Both parameters were affected by the fining procedure following a similar pattern but with some differences. In this sense, the addition of GSP in the advanced stages of the vinification induced a decrease in the total phenolic and flavanol contents in all Syrah red wines. Thus, these two variables have been analyzed as total phenolics and total flavanols adsorption. The adsorption experienced by the fined wines varied from 36.57 to 171.01 mg gallic acid/L and 6.67 to 37.41 mg catechin/L for TPC and TFC, respectively (Table 1).

The F values in this model for pH(linear) (F = 132.70, *p* < 0.01), pH(quadratic) (F = 10.54, *p* < 0.05), ethanol(linear) (F = 371.06 (*p* < 0.001)) and ethanol(quadratic) (F = 74.60, *p* < 0.01) show the relevance of these factors, both in their linear and quadratic forms, in the adsorption of TPC. These findings agree with the observation previously described by Kang et al. [27] related to the relevance of these factors for reducing total phenolic compounds using potato protein during fining of Cabernet Sauvignon wines. According to the linear term, a decrease in pH and ethanol content leads to an increase in adsorption of phenolic compounds during fining (Figure 1b). However, a notable influence of the ethanol content, with respect to pH, on the response could be appreciated according to the higher sum of the square term (SS) in the model (SS = 28223.5 vs. SS = 9071). The highest differences are observed between wines with 11 or 13% (*v*/*v*) ethanol and wines with 15% (*v*/*v*) at low pH values. The significant relevance of the square term of pH and ethanol resulted in a parabolic trend in total phenolic adsorption. This reflects efficient adsorption at low values of pH and ethanol content; however, below a certain level not considered in this study, an inverse behavior is expected. Once more, the influence of the quadratic term of ethanol content is greater, so the change in trend predicted by the model will be more pronounced for decreases in the wine ethanol content than pH, as can be seen in Figure 1b. However, the values from which the change could occur are unusual in wines.

Related to TFC, only the linear term of the ethanol content was relevant in the adsorption of flavanol compounds (F = 14.97, *p* < 0.05), showing a negative effect on the response variable (Figure 1c). Although pH has an important influence in the same sense as ethanol, it was not relevant for this variable in the developed model (F = 9.59, *p* = 0.053). Taking this into account, the most notable differences in the adsorption of total flavanols were observed depending on the ethanol content of the wines, especially between wines with 11 or 13% (*v*/*v*) and wines with 15% (*v*/*v*). The bitterness and astringency of wine are strongly conditioned by the flavanol content, and therefore protein-based fining agents can determine some declines in wine astringency and bitterness due to its interaction with tannins [11]. According to the results obtained in this work, in addition to the fining agent, the wine ethanol content could be considered a parameter of interest for winemakers to obtain wines with smoother taste sensations. However, it is not useful to modify the pH for this purpose.

#### 3.1.3. Impact on Antioxidant Activity

**Figure 2:**
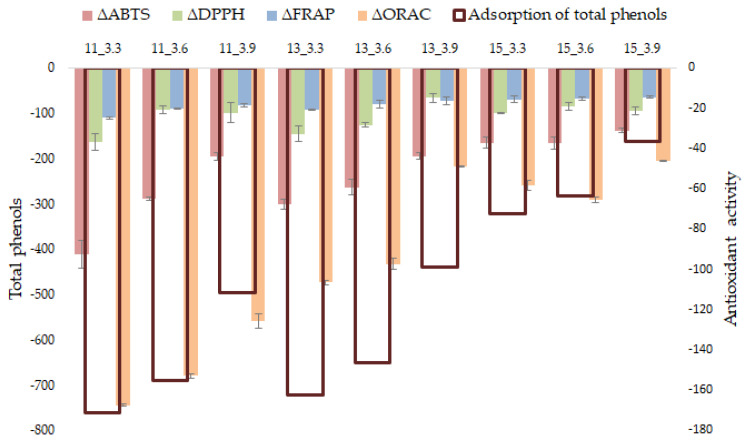
Influence of wine pH and ethanol content on adsorption of total phenolics (mg Eq gallic acid/L) and antioxidant activity modification (mg EqTE/L for ABTS, DPPH and ORAC, mg Eq AAE/L for FRAP) of wines fining with GSP.

Due to the complexity of the red wine matrix, the antioxidant activity has been assessed in different ways using four different spectrophotometric methods. The capacity of red wines to remove free radicals was evaluated using DPPH and ABTS assays. The ability to reduce a ferricyanide/Fe^3+^ complex was quantified using the FRAP assay, while the neutralization capacity of the peroxyl radical was evaluated using the ORAC assay. Modifications in the antioxidant capacity of red wines fined with GSP are shown in Figure 2. As can be observed, after the fining treatment, all red wines showed a reduction in their antioxidant capacity regardless of the method used, and the antioxidant activity was in accordance with TPC. Thus, in general, red wines with the greatest decrease in TPC showed the highest decrease in antioxidant activity. Univariate linear regression analysis was applied to explore the influence of TPC on antioxidant activity reaching correlation coefficients of 0.943, 0.940, 0.962, and 0.963 for DPPH, ABTS, FRAP, and ORAC, respectively. The key role of phenolic compounds in the antioxidant activity in different matrices has also been described [38,39,40].

In order to assess the relative effect of pH and ethanol content on antioxidant activity, ANOVA of the 3^2^ full factorial design was performed for all spectrophotometric methods used in this study (Table 1). Similar results were obtained for all the assays, except DPPH, for which none of the factors analyzed turned out to be relevant. For the other, a significant influence of the pH and ethanol content, in linear form, on the antioxidant activity was found. Following the trend observed in the parameters previously analyzed, a notable influence of the ethanol content in relation to the pH was found. The results also revealed the positive influence of the pH x ethanol interaction on the antioxidant activity determined by ABTS and FRAP assays. The most notable decreases in antioxidant activity were found in wines with low ethanol and pH values (ABTS: 10.96%; DPPH: 7.12%; FRAP: 4.97%; ORAC: 21.67%). Even in this case, the impact on antioxidant activity can be considered acceptable in comparison with the benefits that fining treatment can provide in other sensorial attributes [41].

#### 3.1.4. Impact on Chromatic Characteristics

The color of red wines is the first attribute perceived by the consumer and, therefore, one of the most decisive factors for the consumer’s preferences. Among the benefits of clarification with fining agents, in addition to the increase in limpidity, color stabilization is notable because of the reduction of colloidal phenolic compounds related to the wine color and involved in oxidation processes. However, excessive clarification in wines with low phenolic content, as usually occurs in warm climate wines, can be problematic. In this sense, chromatic characteristics of the wines fining with GSP have been evaluated. Table 1 shows the differences for the CIELAB colorimetric parameters (ΔL*, Δa*, Δb*, ΔC*_ab_, and Δh_ab_) of each fined red wine with respect to its control and the relative effect of the pH and ethanol content according to the ANOVA of the experimental design. As can be seen, fining induced color changes in red wines in both qualitative (h_ab_) and quantitative (L* and C*_ab_) aspects. In general, the fined wines showed a slight increase in the lightness (L*) and chroma (C*_ab_) values, while a decrease in the hue values (h_ab_) was observed. This fact is translating into clearer, more chromatic intensity and more red–blue hues wines than the control. This trend toward more bluish tonalities was also confirmed by the decrease in the b* values in the redness region of the CIELAB space, which can be appreciated in most of the wines analyzed. However, no significant influence of pH and ethanol content on the wine’s chromatic characteristics changes was found (*p* > 0.05). Only wine with pH 3.3 and 11% (*v*/*v*) ethanol showed significant differences in colorimetric attributes. In this sense, an increase of 9% in the wine color intensity (C*_ab_ = 22.71 vs. 20.89) and a reduction of 41% in the hue (h_ab_ = 2.99 vs. 4.11) were observed for this wine after fining. Moreover, it is the only wine that experienced a lightness decrease after clarification.

The color changes between each wine and its control (not treated with GSP) have been quantified using the color difference (ΔE*_ab_). Consistent with the results obtained in other clarification assays carried out on wines in advanced stages of vinification [9,42], a generally low impact on color was observed for all wines. The color differences ranged from 0.09 to 2.11. Considering that only ΔE*_ab_ higher than three CIELAB units can be perceived by the human eye, the color changes experienced by all experimental wines are visually imperceptible, even for wines with the lowest pH levels and ethanol contents.

### 3.2. Grape Seed Protein as an Alternative to Commercial Protein Fining Agents

Based on the results obtained in the experimental design developed in the first part of this study, where the lowest values of pH and ethanol content allow to achieve the best levels of clarification, wines with pH 3.3 and 11% (*v*/*v*) ethanol have been used to evaluate the potential of GSP, obtained as a winery by-product, as an alternative to other vegetal proteins (isolated from potato and pea) commonly used in winemaking. Attention has focused on clarification and chromatic characteristics.

As can be observed in Table 4, the effectiveness of the clarification varied significantly according to the fining agent. The highest percentages of clarification were observed in wines infused with potato protein (13.40%), followed by GSP (11.47%), while the effect of pea protein was practically negligible in the analyzed wines (1.42%). Even so, taking into account the protein content of the fining agents (79.92%, 72.54%, and 55.35% for potato, pea, and grape seed isolate, respectively), GSP has been shown to be similar in efficacy to potato protein and higher than pea protein.

Concerning chromatic characteristics, all fining agents significantly (*p* < 0.05) modified the CIELAB color parameters of the wines (L*, a*, b*, C*_ab_, and h_ab_) compared to their control (Table 4). Overall, fine wines showed lower values of lightness and hue (L* and h_ab_) and higher chroma values (C*_ab_) than the control, which translates into darker wines with more chromatic intensity and red–blue hues. Only wines fined with pea protein showed a slight increase in lightness. This colorimetric attribute was not significantly (*p* < 0.05) different with respect to control in fine wines with potato and pea protein. The degree of color changes varied depending on the fining source. In this sense, grape seed and potato protein produced the highest impact on the quantitative attribute of color by decreasing the lightness (L* = 77.80 and 78.52 vs. 78.62 in control) and increasing the intensity of the color (C*_ab_ = 22.71 and 22.23 vs. 20.89 in control). For both L* and C*_ab_, the differences between grape seed and potato protein turned out to be significant with respect to the effect of the pea protein (*p* < 0.05). These fining agents also had a significant impact from a qualitative point of view (h_ab_), showing a hue shift toward more bluish red hues in the redness region of the CIELAB space. However, the greatest influence on the tonality of wines was produced by pea protein (h_ab_ = 2.99, 2.83, and 2.34 for GSP, potato protein, and pea protein, respectively, vs. 5.11 in the control). The addition of GSP allows the clarification of wines in advanced stages of vinification without significant loss of chromatic wine characteristics.

## 4. Conclusions

In this study, the impact of wine characteristics (pH and ethanol content) on the fining efficacy of grape seed protein in warm-climate red wines has been evaluated. The most noticeable clarifying effects were observed for the experimental wines with lower pH and ethanol content. The control of these factors will make it possible to modulate the main organoleptic properties of the wines, also avoiding the addition of large amounts of fining agents and, then, its associated drawbacks. In this sense, the ethanol content has proven to be the most relevant factor for the different parameters analyzed. Therefore, the findings obtained in this work imply greater versatility and efficiency for wineries during the winemaking process.

Furthermore, grape seed protein has been shown to arise as a promising fining alternative to other plant-based proteins that are commonly used in winemaking, such as potato or pea protein. The effects on clarification and color quality have been found to be comparable to those of potato protein and significantly better than those of pea protein. In addition to being endogenous to grapes and being a non-allergenic agent, the use of vinification by-products as an alternative to the use of other stabilizers, including plant-based proteins, represents a sustainable alternative for the wine industry, reducing the contamination effects of their wastes and increasing their value by conversion into useful by-products. In future studies, it would be interesting to test different red wines, wines in earlier stages of vinification, as well as other factors and their influence on other variables directly related to the sensory quality of wines.

## Figures and Tables

**Table 1:** Description of experimental Syrah red wines according to the wine pH and ethanol content (% *v*/*v*) factors and their levels in the full factorial design.

	Coded Factors		Uncoded Factors	
Sample	x_1_ (pH)	x_2_ (Ethanol Content)	x_1_ (pH)	x_2_ (Ethanol Content)
GSP_11_3.3	−1	−1	3.3	11
GSP_11_3.9	1	−1	3.9	11
GSP_13_3.3	−1	0	3.3	13
GSP_11_3.6	0	−1	3.6	11
GSP_13_3.9	1	0	3.9	13
GSP_15_3.6	0	1	3.6	15
GSP_13_3.6	0	0	3.6	13
GSP_15_3.3	−1	1	3.3	15
GSP_15_3.6	1	1	3.6	15

**Table 2:** Analytical data of base Syrah red wine (Means ± SD, n = 3).

pH	Ethanol Content (% *v*/*v*)	Reducing Sugars (g/L)	Total Acidity (g/L as Tartaric Acid)	Volatile Acidity (g/L as Acetic Acid)
3.45 ± 0.03	12.85 ± 0.16	1.27 ± 0.11	5.43 ± 0.09	0.52 ± 0.04

**Table 3:** Influence of wine pH (3.3, 3.6 and 3.9) and ethanol content (11, 13 and 15 % *v*/*v*) on clarification (%, n = 3), adsorption of total phenolics (mg Eq gallic acid/L, n = 3), adsorption of total flavanols (mg Eq catechin/L, n = 3), antioxidant activity modification (mgTE/L for ABTS, DPPH and ORAC, mgAAE/L for FRAP, n = 3) and colorimetric characteristics modification (mean, n = 3) induced by fining process with grape seed protein.

				Antioxidant Activity				Colorimetric Characteristics				
	Clarification	Adsorption of Total Phenolics	Adsorption of Total Flavanols	ΔABTS	ΔDPPH	ΔFRAP	ΔORAC	ΔL*	Δa*	Δb*	ΔC*ab	Δhab
GSP_11_3.3	11.47 ^e^	171.04 ^a^	37.41 ^c^	−411.00 ^d^	−163.54 ^c^	−110.43 ^b^	−744.46 ^g^	−0.814 ^a^	1.813 ^a^	−0.709 ^a^	1.825 ^a^	−2.117 ^a^
GSP_11_3.6	9.19 ^d^	154.96 ^a^	22.31 ^a,b^	−288.29 ^c^	−92.07 ^a,b^	−90.16 ^a,b^	−679.79 ^f^	0.516 ^c^	−0.296 ^b^	0.130 ^c^	0.451 ^b^	−0.211 ^e,f^
GSP_11_3.9	7.53 ^c^	111.46 ^d,e^	23.72 ^b^	−195.17 ^a,b^	−99.42 ^a,b,c^	−81.68 ^a,b^	−558.88 ^e^	0.057 ^b^	0.072 ^d,e^	−0.088 ^b,c^	0.065 ^e,f^	−0.253 ^f^
GSP_13_3.3	8.65 ^c,d^	162.34 ^a^	32.37 ^c^	−301.57 ^c^	−145.08 ^b,c^	−92.41 ^a,b^	−473.42 ^d^	0.533 ^c^	−0.075 ^c,d^	−0.058 ^b,c^	0.022 ^c,e^	−0.046 ^d,e^
GSP_13_3.6	7.14 ^b,c^	146.43 ^a^	20.59 ^a,b^	−263.68 ^b,c^	−125.85 ^a,b,c^	−79.38 ^a^	−432.97 ^d^	0.109 ^b^	0.118 ^e^	−0.038 ^c^	0.150 ^f^	−0.092 ^d,e,f^
GSP_13_3.9	5.87 ^a,b^	99.04 ^c,d^	19.87 ^a,b^	−194.04 ^a,b^	−66.35 ^a^	−72.07 ^a^	−217.59 ^a,b^	0.546 ^c^	0.110 ^d,e^	−0.365^b^	0.108 ^e,f^	−0.913 ^b^
GSP_15_3.3	5.60 ^a,b^	72.16 ^b,c,d^	16.02 ^a^	−164.8 ^a^	−98.65 ^a,b,c^	−68.99 ^a^	−258.94 ^b,c^	0.356 ^c^	0.118 ^e^	−0.030 ^c^	0.158 ^f^	−0.014 ^c,d^
GSP_15_3.6	5.16 ^a^	63.52 ^b,c^	16.36 ^a^	165.56 ^a^	−85.31 ^a,b^	−67.54 ^a^	−290.20 ^c^	0.044 ^b^	−0.023 ^c,d,e^	−0.003 ^c^	−0.056 ^c,d^	0.149 ^c^
GSP_15_3.9	5.04 ^a^	36.57 ^b^	6.67 ^a^	−137.86 ^a^	−93.91 ^a,b,c^	−64.27 ^a^	−204.85 ^a^	0.381 ^c^	−0.127 ^b,c^	−0.024 ^c^	−0.143 ^d^	−0.131 ^d,e,f^
pH (L)	***	**	ns	**	ns	**	*	ns	ns	ns	ns	ns
pH (Q)	ns	*	ns	ns	ns	ns	ns	ns	ns	ns	ns	ns
Ethanol (L)	***	***	*	***	ns	***	**	ns	ns	ns	ns	ns
Ethanol (Q)	ns	**	ns	ns	ns	ns	ns	ns	ns	ns	ns	ns
pH*ethanol	**	ns	ns	*	ns	*	ns	ns	ns	ns	ns	ns

Different letters in the same column indicate statistical differences (Tukey test, α = 0.05). For each parameter, Δ has been calculated as the difference between the value obtained for the experimental wine and the value obtained for its control. (L): linear term; (Q): Quadratic term; *** *p* < 0.001; ** *p* < 0.01: * *p* < 0.05; ns: no significant differences.

**Table 4:** Clarification (%, mean ± SD) and chromatic parameters (mean ± SD) of Syrah red wines with pH 3.3 and 11% (*v*/*v*) ethanol content before (control wine) and after fining treatment with grape seed, potato or pea proteins.

	Control Wine	Grape Seed Protein	Potato Protein	Pea Protein
Clarification	-	11.47 ± 0.71 ^a^	13.40 ± 0.37 ^b^	1.42 ± 0.48 ^c^
L*	78.62 ± 0.19 ^a^	77.80 ± 0.20 ^b^	78.52 ± 0.11 ^a^	78.76 ± 011 ^a^
a*	20.80 ± 0.04 ^a^	22.62 ± 0.14 ^b^	22.17 ± 0.06 ^c^	21.71 ± 0.05 ^d^
b*	1.88 ± 0.12 ^a^	1.17 ± 0.06 ^b^	1.12 ± 0.06 ^b^	0.89 ± 0.03 ^b^
C*_ab_	20.89 ± 0.05 ^a^	22.71 ± 0.14 ^b^	22.23 ± 0.06 ^c^	21.73 ± 0.05 ^d^
h_ab_	5.11 ± 0.31 ^a^	2.99 ± 0.31 ^b^	2.83 ± 0.14 ^b^	2.34 ± 0.09 ^c^

Different letters in the same row indicate significant differences (Tukey test α = 0.05).

## Data Availability

Data are contained within the article.

## Abbreviations

GSP: Grape seed protein; TPC, total phenol content; TFC, total flavanol content; DMACA, 4-(Dimethylamino)cinnamaldehyde; DPPH, 2,2-difenil-1-picrilhidrazilo; ABTS, 2,2′-azino-bis(3-ethylbenzothiazoline-6-sulfonic acid)); FRAP, ferric reducing antioxidant power; ORAC, Oxygen Radical Absorbance Capacity; AAPH, 2.2′-azobis(2-methylpropionamidine) dihydrochloride; PBS phosphate buffered saline; ANOVA, Univariate analyses of variance.
